# Should we standardize PhD training in neuroscience?

**DOI:** 10.1093/braincomms/fcad028

**Published:** 2023-03-02

**Authors:** Tara L Spires-Jones

**Affiliations:** Edinburgh, UK

## Abstract

Our editor discusses the need for standardization of neuroscience PhD training in the UK.

## Graphical Abstract

**Graphical Abstract:**
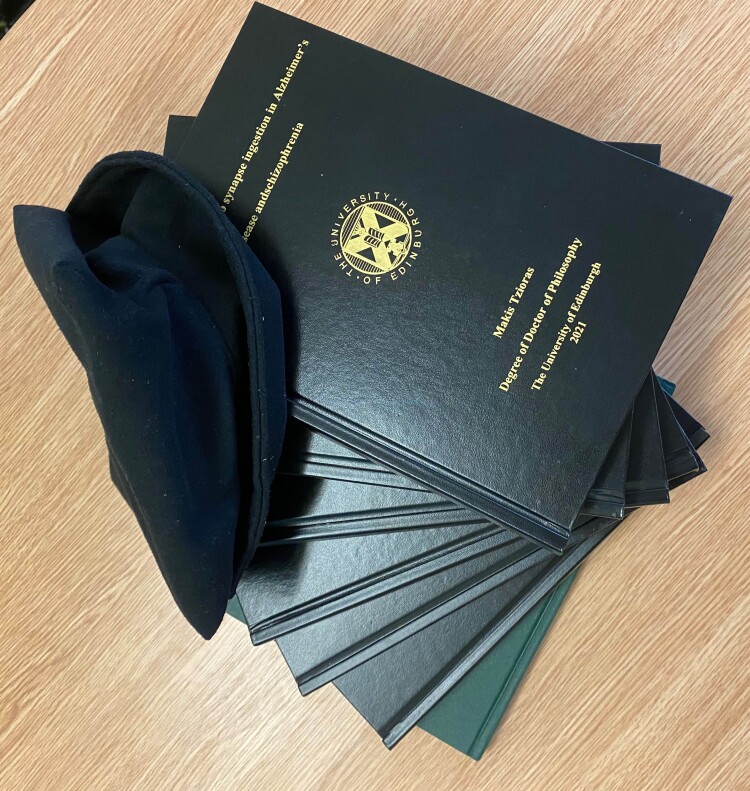

Welcome to volume 5 issue 2 of Brain Communications. In our last editorial,^1^ we launched an early career researcher paper prize, and I’m delighted to announce the winner: George Thomas, for his paper ‘Changes in both top-down and bottom-up effective connectivity drive visual hallucinations in Parkinson’s disease’.^2^ Many congratulations!

I have been thinking a lot lately about early career researchers, and in particular about doctoral training in neuroscience. The doctor of philosophy (PhD or DPhil), using the broad meaning of philosophy ‘love of wisdom’, originated in the 1800s in Humboldt University in Germany and spread from there to the US, UK, then the rest of the world.^3^ Since its origins, the main requirements for a PhD have remained similar—to produce new knowledge through research and defend a thesis or dissertation describing the research. Although the main objectives of a PhD are shared throughout the world, there is substantial variation in the actual training received by PhD students. ^4^ In fact, we do not know the extent of variability in PhD training. Powell and Green wrote in their book on doctoral training ‘Comparative study seems to be almost completely lacking at the level of the doctorate’. ^3^ Even within the UK (where *Brain Communications* is based), there is considerable variability between the training experienced by neuroscience PhD students. Many students spend 3 years in a research project without any formal taught requirements, whether or not they had any previous training in the wider neuroscience field. In some 4-year PhD programmes, students receive a year of formal coursework and/or lab rotations before starting their projects, but there is not consistency across the country around which aspects of neuroscience are taught. Even within the same institution, the transient nature of the funding for these 4-year programmes means that we often have to completely re-invent training plans for each new grant application.

Why, you may ask, does this matter? Neuroscience is a broad field and our early career researchers have diverse interests, so does it matter if training varies widely? I argue that some level of consistency in neuroscience PhD training would benefit both the research field and the students. In particular, a solid grounding in best practice for experimental design, data analysis, and critical thinking are crucial. Even if a student is not running traditional ‘wet lab’ experiments during their PhD, the ability to critically appraise experiments in the field is a key skill. This training helps students succeed in their projects and benefits the field by enhancing credibility. Students will be more likely to design robust, replicable experiments with appropriate training. Similarly, training in key areas of neuroscience and relevant methodologies helps students by broadening their research horizons and helps produce well-rounded graduates who are ready to contribute to many areas of research and teaching.

In my view, we need to think about the future of neuroscience PhD training in the UK to provide the best start for our students and to maintain world-class neuroscience research outputs in the country. I do not have concrete plans about the best way to do this and would love to hear what you all think.

The cover of this issue comes from Want *et al*. ^5^ and shows DiOlistic labelling of mouse retinal ganglion cells which was used to demonstrate that brain-derived neurotrophic factor (BDNF) released from blood platelets prevents dendritic atrophy of lesioned adult CNS neurons.
